# The effects of AICAR and rapamycin on mitochondrial function in immortalized mitochondrial DNA mutator murine embryonic fibroblasts

**DOI:** 10.1242/bio.033852

**Published:** 2018-09-03

**Authors:** Vedad Delic, Kenyaria Noble, Sandra Zivkovic, Tam-Anh Phan, Christian Reynes, Yumeng Zhang, Oluwakemi Phillips, Charles Claybaker, Yen Ta, Vinh B. Dinh, Josean Cruz, Tomas A. Prolla, Patrick C. Bradshaw

**Affiliations:** 1 Department of Neurology, Center for Neurodegeneration and Experimental Therapeutics, University of Alabama Birmingham School of Medicine, Birmingham, AL 35233, USA; 2 Department of Cell Biology, Microbiology and Molecular Biology, University of South Florida, Tampa, FL 33620, USA; 3 Department of Internal Medicine, University of South Florida, Tampa, FL 33606, USA; 4 University of South Florida College of Medicine, Department of Molecular Pharmacology and Physiology, Tampa, FL 33612, USA; 5 Department of Genetics and Medical Genetics, University of Wisconsin-Madison, Madison, WI 53706, USA; 6 Department of Biomedical Sciences, James H. Quillen College of Medicine, East Tennessee State University, Johnson City, TN 37614, USA

**Keywords:** mTOR, AMP kinase, Aging, Mitochondria, Rapamycin, Pyruvate addiction

## Abstract

Mitochondrial DNA mutations accumulate with age and may play a role in stem cell aging as suggested by the premature aging phenotype of mitochondrial DNA polymerase gamma (POLG) exonuclease-deficient mice. Therefore, E1A immortalized murine embryonic fibroblasts (MEFs) from POLG exonuclease-deficient and wild-type (WT) mice were constructed. Surprisingly, when some E1A immortalized MEF lines were cultured in pyruvate-containing media they slowly became addicted to the pyruvate. The POLG exonuclease-deficient MEFs were more sensitive to several mitochondrial inhibitors and showed increased reactive oxygen species (ROS) production under standard conditions. When cultured in pyruvate-containing media, POLG exonuclease-deficient MEFs showed decreased oxygen consumption compared to controls. Increased AMP-activated protein kinase (AMPK) signaling and decreased mammalian target of rapamycin (mTOR) signaling delayed aging and influenced mitochondrial function. Therefore, the effects of 5-aminoimidazole-4-carboxamide ribonucleotide (AICAR), an AMPK activator, or rapamycin, an mTOR inhibitor, on measures of mitochondrial function were determined. Rapamycin treatment transiently increased respiration only in WT MEFs and, under most conditions, increased ATP levels. Short term AICAR treatment transiently increased ROS production and, under most conditions, decreased ATP levels. Chronic AICAR treatment decreased respiration and ROS production in WT MEFs. These results demonstrate the context-dependent effects of AICAR and rapamycin on mitochondrial function.

## Abstract (precis)

**Summary:** A novel mitochondrial DNA mutator murine embryonic fibroblast cell line was created and the effects of the anti-aging compounds rapamycin and AICAR on energy metabolism in these cells was determined.

## INTRODUCTION

Several lines of evidence suggest the involvement of mitochondrial dysfunction in the aging process (Dai et al., 2012; Park and Larsson, 2011; Prolla and Denu, 2014). With aging, most eukaryotic organisms accumulate mitochondrial DNA (mtDNA) point mutations and/or deletions (Kazachkova et al., 2013; Melov et al., 1995; Yui et al., 2003). Evidence indicates that the mtDNA base substitution mutations in aged cells are primarily caused by DNA polymerase gamma (POLG) errors early in development that clonally expand over the lifespan (Larsson, 2010). But the cause of the mtDNA deletions present in aged cells is less clear. There is evidence that oxidative stress contributes to mtDNA deletion formation (Zsurka et al., 2018) and that selective replication of deleted mtDNA molecules allows them to outcompete wild-type (WT) mtDNA molecules in just a few years. This may occur because mtDNA molecules with select mtDNA deletions elude feedback inhibition of the coupled processes of mtDNA transcription and replication (Kowald and Kirkwood, 2018). With aging, mtDNA mutations build up to deleterious levels in individual cells in specific tissue regions, such as muscle fibers (McKenzie et al., 2002), neurons in the substantia nigra region of the brain (Kraytsberg et al., 2006), or in stem cells such as those present in the colonic crypts (McDonald et al., 2006). The mitochondrial dysfunction that occurs in stem cells of POLG exonuclease-deficient (also called mtDNA mutator) mice is likely responsible for many of the premature aging phenotypes that arise in this model (Ahlqvist et al., 2012), although the stem cell alterations were somewhat distinct from those that occur during normal mouse aging (Norddahl et al., 2011). The mitochondrial dysfunction in stem cell niches from mtDNA mutator mice is likely facilitated by a decline in mitophagy in these cells (Li-Harms et al., 2015). Since endurance exercise rescued the premature aging phenotype and oxidative damage to mtDNA without substantially changing the mtDNA mutation load in these mice, mitochondrial RNA polymerase errors caused by oxidative damage to mtDNA is a potential mechanism driving the premature aging phenotype (Safdar et al., 2016a). Endurance exercise increases mitochondrial biogenesis and mtDNA copy number, which may also be partly responsible for protection (Jiang et al., 2017; Safdar et al., 2011).

If mtDNA mutations occurring in young-to-middle aged organisms contribute to aging, then therapies that stimulate mitochondrial turnover through increased mitophagy of organelles with mutant mtDNA and increased biogenesis of organelles with normal mtDNA throughout a lifespan may prove effective at delaying this process. Two methods for inducing autophagy and mitophagy are activation of the AMP-dependent protein kinase (AMPK) signaling pathway and inhibition of the mechanistic target of rapamycin (mTOR) signaling pathway (Meley et al., 2006).

AMPK is one of the main metabolic sensors responding to increases in ADP and AMP levels in the cell (Hardie, 2011; Steinberg and Kemp, 2009). But AMPK can also be activated by Ca^2+^ signaling (Hawley et al., 2005) or increased levels of the glycolytic intermediate fructose 1,6-bisphosphate (Lin and Hardie, 2018). AMPK is a conserved heterotrimer (αβγ) and overexpression of an alpha subunit has been shown to extend the lifespan in *Caenorhabditis elegans* (Apfeld et al., 2004). AMPK activates glucose uptake, glycolysis, fatty acid uptake and fatty acid beta-oxidation, in addition to inhibiting the biosynthesis of glycogen, fatty acids, sterols, triglycerides and phospholipids (Hardie and Ashford, 2014). AMPK activation has been shown to improve skeletal muscle energy metabolism, possibly through stimulation of mitochondrial biogenesis, while decreasing the phosphorylation and activity of mTOR to stimulate autophagy (Abbas and Wink, 2010; Egan et al., 2011; Wang et al., 2010). There is an aging-related decline in the ability to activate AMPK (Reznick et al., 2007), which could contribute to the impaired mitochondrial electron transport chain (ETC) function and impaired autophagy (Rajawat et al., 2009) occurring in aged tissues.

mTOR is a kinase that forms two distinctive complexes, mTORC1 and mTORC2. mTORC1 activity is stimulated by increased oxygen levels, amino acids, energy levels and nutrient availability to promote increased metabolism and cell cycle progression (Halloran et al., 2012; Laplante and Sabatini, 2012). mTORC1 activation leads to increased protein synthesis, cell growth, cell proliferation and cell motility and an inhibition of autophagy (Kim et al., 2011). mTORC1 activity is inhibited by nutrient stress such as calorie restriction (CR), and is potently inhibited by the compound rapamycin (Laplante and Sabatini, 2012), although long term rapamycin treatment also inhibits mTORC2 function (Schreiber et al., 2015). Decreasing mTOR activity with rapamycin has been shown to increase lifespan in mice (Harrison et al., 2009).

Viral E1A immortalized mtDNA mutator and WT cell lines were established for the experiments performed here to more easily study how rapamycin and the AMPK activator AICAR affect cellular energy metabolism in mitotic cells with decreased mitochondrial ETC activity, since accumulating evidence suggests that mitochondrial dysfunction in mitotic stem cells may play a role in the aging process (Ahlqvist et al., 2012; Baines et al., 2014; Wahlestedt et al., 2014). The effects of AICAR and rapamycin on energy metabolism in cells, such as certain types of stem cells, which primarily generate ATP by glycolysis instead of by oxidative phosphorylation, is not well understood. Therefore, experiments were performed to examine how varying glucose and pyruvate levels in the culture media altered the effects of AICAR and rapamycin on mitochondrial function and cellular ATP levels. While characterizing the E1A immortalized WT and mtDNA mutator cell lines we identified that the addition of pyruvate to the culture medium stimulated colony formation and, upon long term culture in the presence of pyruvate, the E1A immortalized cells frequently became addicted to it.

## RESULTS

### E1A immortalized mtDNA mutator MEFs are more sensitive to most mitochondrial inhibitors

**Fig. 1.**
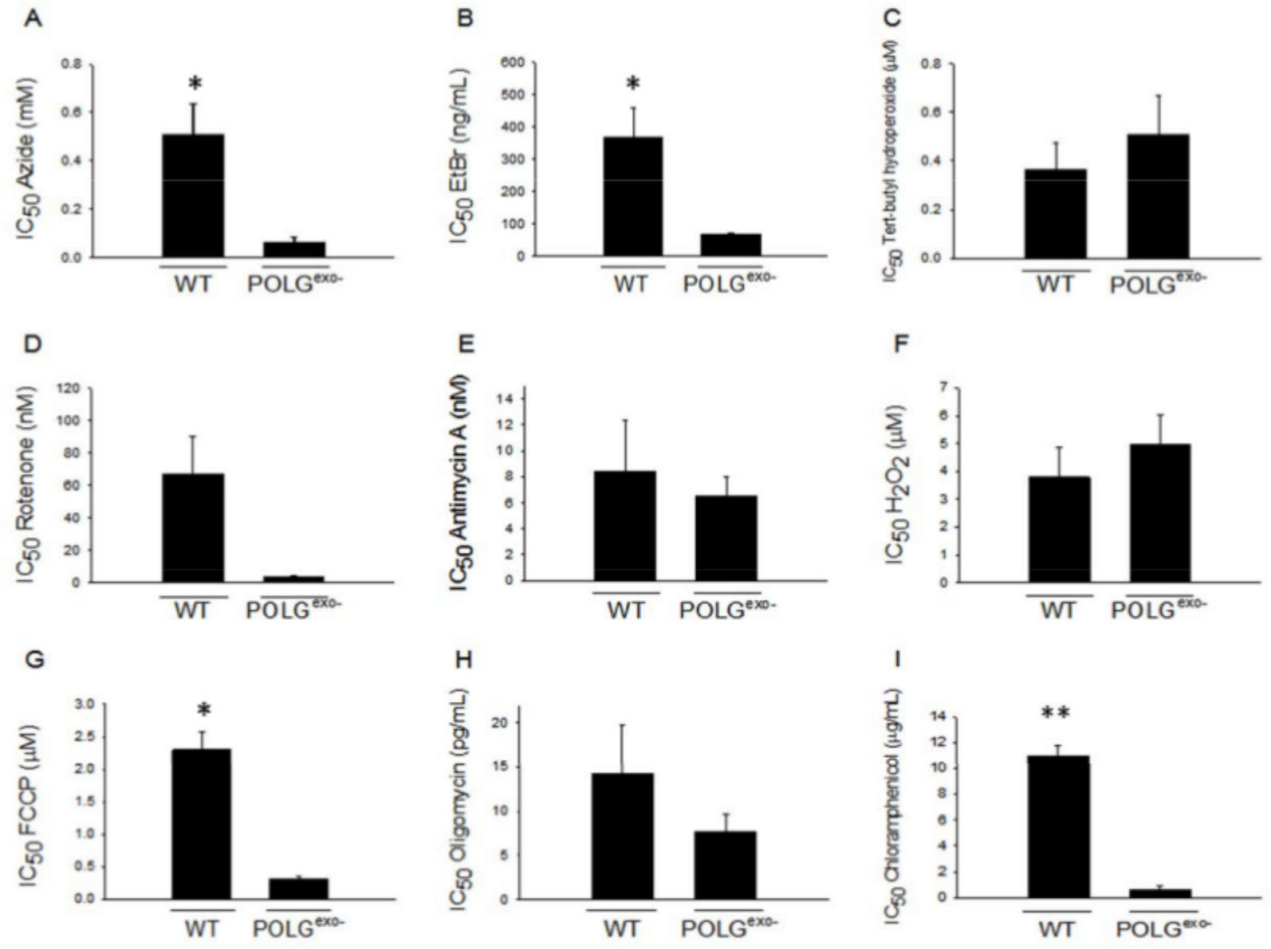
**Sensitivity of WT and MtDNA mutator (POLG^exo−^) MEFs to mitochondrial toxins.** Mitochondrial mutator MEFs were more sensitive than WT MEFs to inhibition of colony formation by azide (*P*=0.025), ethidium bromide (*P*=0.046), FCCP (*P*=0.002) and chloramphenicol (*P*<0.001). A very strong trend for a difference in sensitivity was also found for rotenone (*P*=0.053). Colony counting assays were performed to determine IC_50_ values for (A) azide, (B) ethidium bromide (EtBr), (C) tert-butyl hydroperoxide, (D) rotenone, (E) antimycin A, (F) hydrogen peroxide (H_2_O_2_), (G) FCCP, (H) oligomycin, and (I) chloramphenicol. Experiments were performed with three independently derived E1A immortalized WT and mtDNA mutator clone lines (*n*=3). Bars represent mean±s.e.m. Unpaired *t*-tests were performed with * indicating a *P*-value of <0.05 and ** indicating a *P*-value <0.001.

To establish a new model to study mitotic cell mitochondrial dysfunction, stable adenoviral E1A transfected mtDNA mutator and WT MEF cell lines were generated. The rationale behind the use of the E1A protein to immortalize the MEFs was to prevent the mutation or decline in abundance of p53 (Savelyeva and Dobbelstein, 2011), which commonly occurs during spontaneous transformation of MEFs. Proper p53 function may be important for the identification of therapies that maintain or enhance mitochondrial function with aging as p53 is required for mitochondrial biogenesis (Matoba et al., 2006), the efficient repair of mtDNA (Achanta et al., 2005; de Souza-Pinto et al., 2004) and for full mitochondrial pyruvate dehydrogenase activity (Contractor and Harris, 2012). The mtDNA mutator and WT cell lines were then characterized for the ability to form colonies when grown in the presence of increasing concentrations of mitochondrial inhibitors or oxidants that decrease mitochondrial respiratory function. These studies were performed in part to determine if the new mtDNA mutator cell line retained the same relative insensitivity to oxidative stressors as shown for the primary MEFs (Trifunovic et al., 2005). The results showed that colony formation of mtDNA mutator MEFs were more susceptible than WT MEFs to the ETC complex IV inhibitor azide, the uncoupler FCCP, the mitochondrial protein synthesis inhibitor chloramphenicol and the DNA intercalating agent ethidium which, because of its positive charge, accumulates in mitochondria. There was also a very strong trend for increased sensitivity to the mitochondrial ETC complex I inhibitor rotenone (*P*=0.053). No statistical difference in sensitivity was found for treatment with the ETC complex III inhibitor antimycin A, the mitochondrial F_0_F_1_-ATP synthase inhibitor oligomycin, or the oxidants tert-butyl hydroperoxide or hydrogen peroxide (Fig. 1A-I). There was also no statistical difference for the prevention of colony formation between the two cell lines when the ETC complex III inhibitor myxothiazol was added (data not shown).

### E1A immortalized mtDNA mutator MEFs are not more sensitive to nucleoside analogs

Some nucleoside analog antivirals such as azidothymidine (AZT; a chain terminating nucleoside reverse transcriptase inhibitor used to treat HIV), dideoxycytidine (ddC; a chain terminating nucleoside reverse transcriptase inhibitor formerly used to treat HIV) and fialuridine (FIAU; a non-chain terminating thymidine analog that resulted in lethal hepatotoxicity in a clinical trial for the treatment of hepatitis B) are suspected to cause mitochondrial dysfunction by incorporation into mtDNA (Johnson et al., 2001). Therefore, we tested the toxicity of several nucleoside drugs on WT and mtDNA mutator MEFs. Results showed no significant differences in sensitivity for the prevention of colony formation following the administration of any of the nucleoside analogs including AZT, ddC, FIAU, cytarabine (Ara-C; a non-chain terminating deoxycytidine analog used in chemotherapy) or bromodeoxyuridine (BrdU; a non-chain terminating thymidine analog used experimentally) (Fig. S1), suggesting that the murine POLG exonuclease domain does not play a major role in protecting the cells from the toxicity of the tested nucleoside analogs.

### Pyruvate supplementation reveals a difference in the respiratory rates between E1A immortalized WT and mtDNA mutator MEFs

Because of the known longevity-modulating effects of AMPK and TOR signaling in model organisms and because of the known ability of autophagy to protect mitochondrial function (Wu et al., 2009), a thorough characterization of the effects of AICAR and rapamycin treatment on mitochondrial function and cellular energetics in E1A immortalized mtDNA mutator and WT MEF cell lines was performed. In addition, it was determined if altering the glucose or pyruvate levels in the media affected mitochondrial function or the effects of AICAR and rapamycin on mitochondrial function. To accomplish this task, the immortalized MEFs were cultured in either low (5 mM) glucose media in the presence of 1 mM pyruvate (LGPM), a media that stimulates mitochondrial respiratory function (Weber et al., 2002); in high glucose medium (HGM) (25 mM glucose) that lacks pyruvate; or, for long term experiments in high glucose, pyruvate and uridine-containing medium (HGPUM), a medium where cells can grow in the absence of functional mtDNA (King and Attardi, 1989).

**Fig. 2.**
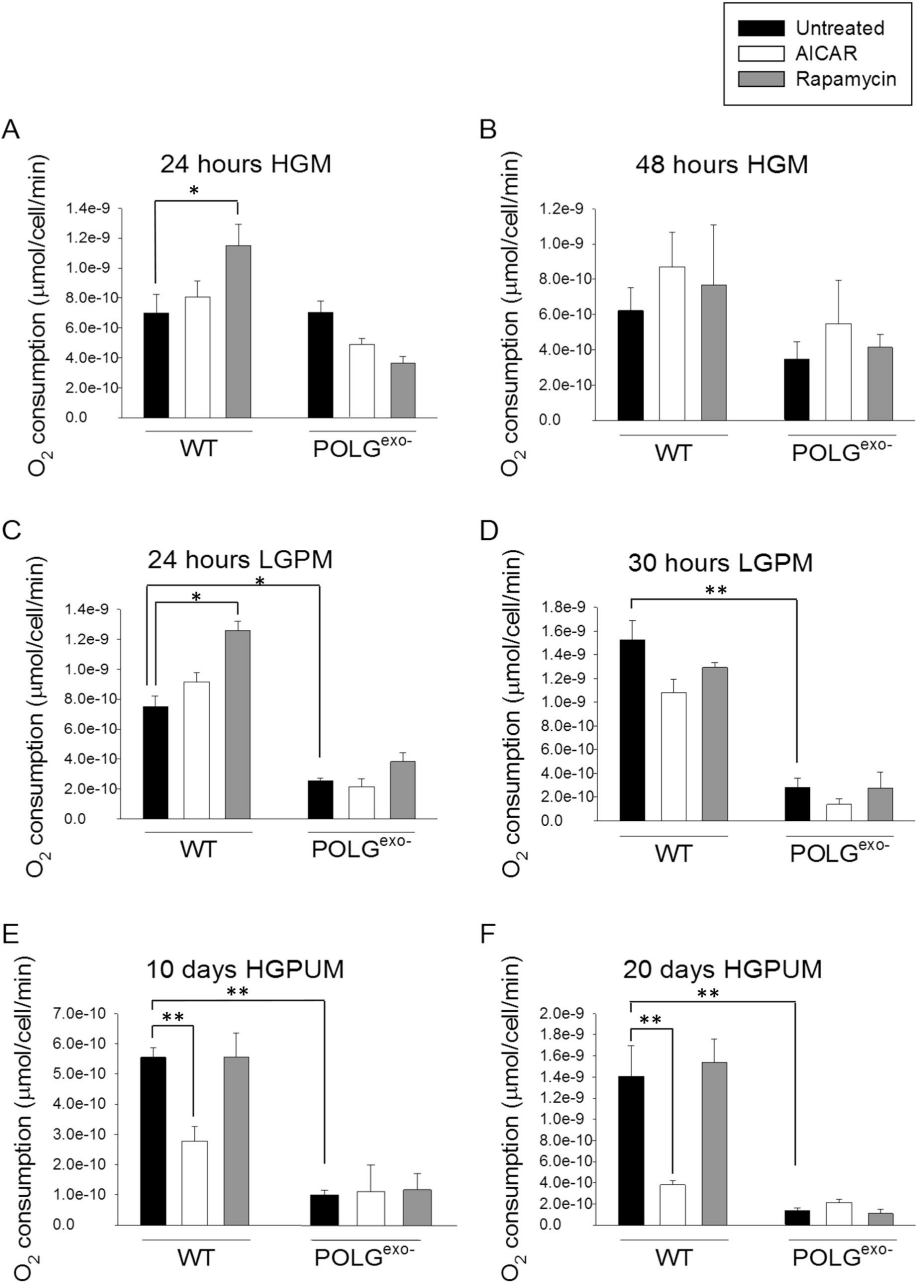
**O_2_ consumption rates of WT and POLG^exo−^ MEFs treated with AICAR or rapamycin.** (A) O_2_ consumption rates after 24 h in HGM. (B) O_2_ consumption rates after 48 h in HGM. (C) O_2_ consumption rates after 24 h in LGPM. (D) O_2_ consumption rates after 30 h in LGPM. (E) O_2_ consumption rates after 10 days in HGPUM. (F) O_2_ consumption rates after 20 days in HGPUM. Rapamycin transiently increases oxygen consumption in WT MEFs at the 24 h time point either in the absence or presence of pyruvate. Respiratory deficits in POLG^exo−^ MEFs were revealed by the LGPM used in panels C and D and the HGPUM used in panels E and F. Experiments were repeated twice using three technical replicates each time. Bars represent mean±s.e.m. Two-way ANOVA with Fisher's LSD post hoc analysis was performed. *P<0.05, **P<0.001.

When cultured in HGM, in contrast to results found with primary MEFs (Trifunovic et al., 2005), no significant difference in the respiratory rates between E1A immortalized WT and mtDNA mutator MEFs was found (Fig. 2A,B). This also greatly contrasts with results using spontaneously transformed MEFs, where mtDNA mutator MEFs were described to respire at only 5% of the rate of WT MEFs (Kukat et al., 2011). However, more than a twofold decline in the respiratory rate was revealed in E1A immortalized mtDNA mutator MEFs when they were switched from HGM to LGPM, while E1A immortalized WT MEFs did not show a significant difference in respiratory rate when cultured in these two types of media. Specifically, mtDNA mutator MEFs showed deficits in respiratory rate after 24 (Fig. 2C) or 30 h (Fig. 2D) of culture in LGPM or after 10 (Fig. 2E), 20 (Fig. 2F) or 29 days (Fig. S2) of culture in HGPUM. There was a slight increase in respiratory rate between the 10 and 20 day time points for WT MEFs cultured in HGPUM, perhaps due to a delayed metabolic adaptation to the pyruvate or uridine present in the culture media. When cultured in HGPUM the mtDNA mutator MEFs showed a greatly decreased respiratory rate on day 10 of culture, the first time point taken, when compared to the rates in HGM. The respiratory rate did not further decline for the next 19 days of culture suggesting that if mtDNA mutations are accumulating when the MEFs are grown in HGPUM, they accumulate quickly and reach a steady state by day 10 of culture.

### Ten or twenty day AICAR treatment decreases the respiratory rate in WT MEFs

Although treatment with the AMPK activator AICAR has been described to increase mitochondrial biogenesis in rodents (Komen and Thorburn, 2014) and human fibroblasts (Golubitzky et al., 2011), we, like others studying mitochondrial function in hepatocytes (Guigas et al., 2007), myocytes (Spangenburg et al., 2013), white adipocytes (Abdul-Rahman et al., 2016) or human fibroblasts (Golubitzky et al., 2011), did not find a significant increase in the respiratory rate following AICAR treatment. This was true using either WT or mtDNA mutator MEFs irrespective of glucose or pyruvate levels in the media or the duration of treatment (Fig. 2A-D). However, we did find that AICAR treatment of WT MEFs for 10 or 20 days cultured in HGPUM led to a strong decrease in respiratory rate (Fig. 2E).

### Rapamycin transiently increases the respiratory rate in WT but not mtDNA mutator MEFs

Rapamycin treatment increased the respiratory rate in WT MEFs after 24 h (Fig. 2A,C) but not after 48 h (Fig. 2B,D) of treatment when cultured in HGM or LGPM. Treatment also did not affect the respiratory rate after 10 (Fig. 2E), 20 (Fig. 2F) or 29 days (Fig. S2) of treatment when cells were cultured in HGPUM. Rapamycin did not significantly increase the respiratory rate under any of these conditions in mtDNA mutator MEFs.

### Reactive oxygen species production increases in mtDNA mutator MEFs in HGM, but decreases following long term culture in HGPUM

**Fig. 3.**
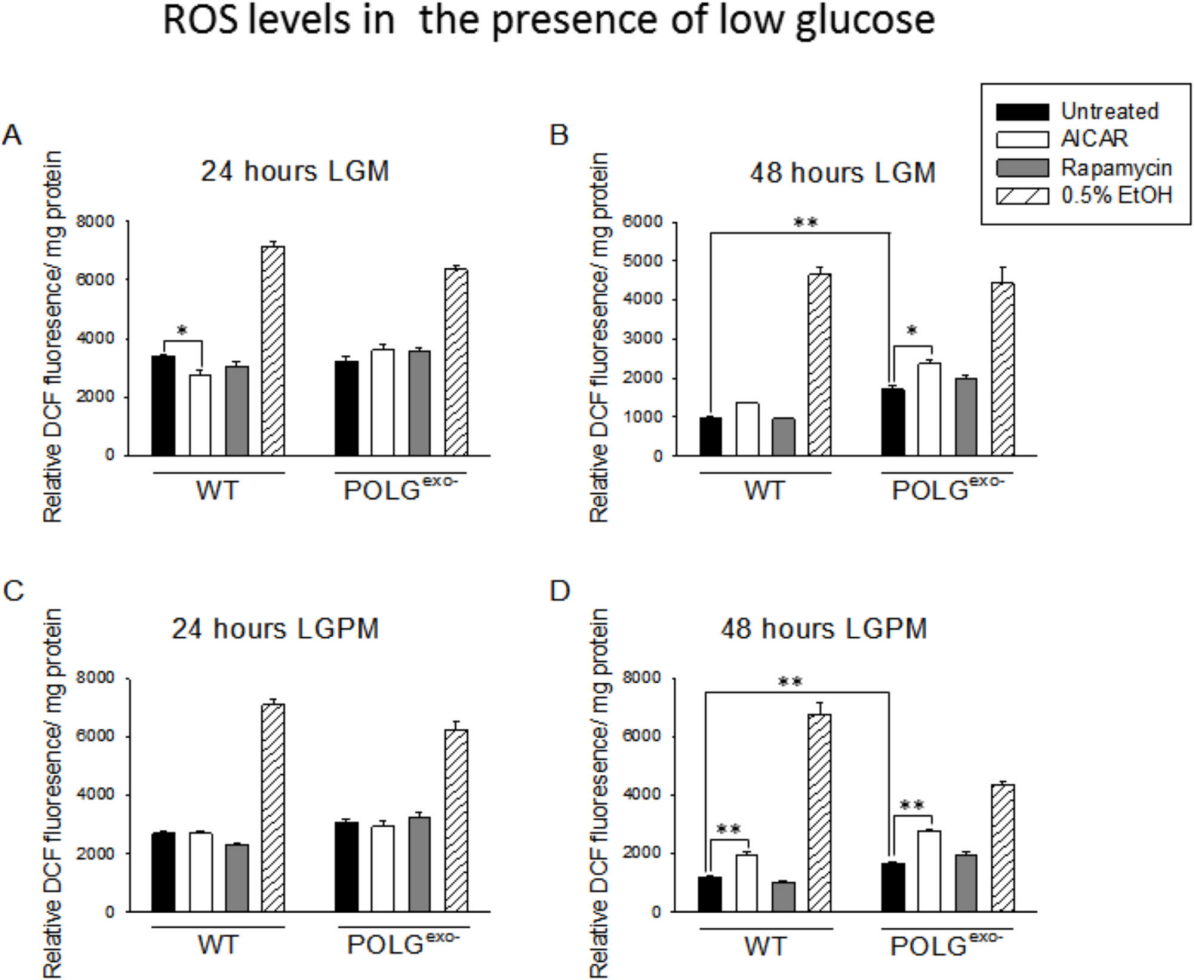
**Effects of AICAR or rapamycin on ROS production of WT and POLG^exo−^ MEFs under low glucose conditions.** Rapamycin treatment in LGM or LGPM had no effect on ROS production. 0.5% ethanol (EtOH) was added as a positive control to induce ROS production. (A) ROS levels after 24 h in LGM. (B) ROS levels after 48 h in LGM. (C) ROS levels after 24 h in LGPM. (D) ROS levels after 48 h in LGPM. Experiments were repeated twice using five technical replicates each time. Bars represent mean±s.e.m. Two-way ANOVA with Fisher's LSD post hoc analysis was performed. *P<0.05, **P<0.001.

**Fig. 4.**
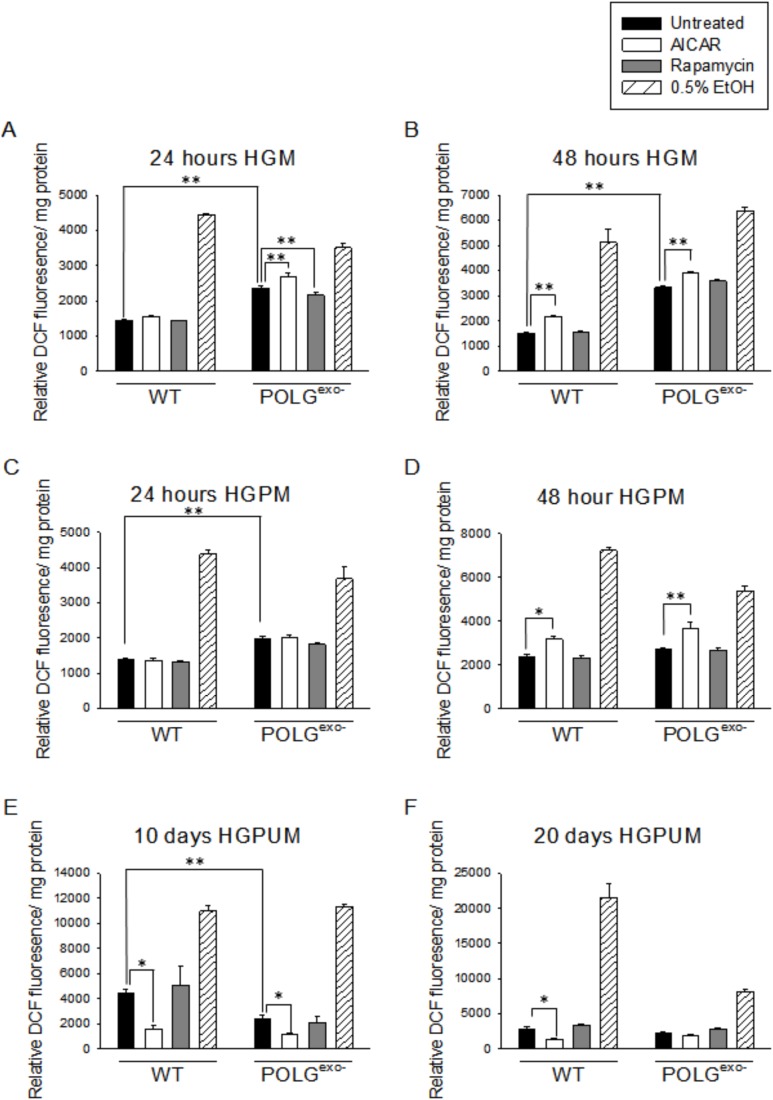
**Effects of AICAR or rapamycin on ROS production of WT and POLG^exo−^ MEFs under high glucose conditions.** 0.5% ethanol (EtOH) was added as a positive control to induce ROS production. (A) ROS levels after 24 h in HGM. (B) ROS levels after 48 h in HGM. (C) ROS levels after 24 h in HGPM. (D) ROS levels after 48 h in HGPM. (E) ROS levels after 10 days in HGPUM. (F) ROS levels after 20 days in HGPUM. Experiments were repeated twice using five technical replicates each time. Bars represent mean±s.e.m. Two-way ANOVA with Fisher's LSD post hoc analysis was performed. *P<0.05, **P<0.001.

These studies were performed in part to determine if the E1A immortalized mtDNA mutator MEFs retained the phenotype of the parental primary MEFs of not increasing reactive oxygen species (ROS) production when cultured in standard HGM (Trifunovic et al., 2005). When cultured in a low glucose medium (LGM) or LGPM, depending upon the duration of treatment and the presence of pyruvate, mtDNA mutator MEFs showed small trends for or small significant increases in ROS production compared to WT MEFs (Fig. 3A-D). In contrast to past results using primary MEFs, large increases in ROS production were observed in the E1A immortalized mtDNA mutator MEFs when they were cultured in standard HGM. The ROS levels declined substantially when the MEFs were cultured in HGPM where pyruvate was present (Fig. 4A-D). In contrast to the results from these short term culture studies, culture for 10 days in HGPUM led to decreased ROS production in mtDNA mutator MEFs compared to WT MEFs (Fig. 4E,F), likely due to the much lower ETC activity present in mtDNA mutator MEFs compared to WT MEFs under these pyruvate-containing culture conditions as indicated by the respiratory studies. Decreased ETC activity can decrease ROS production when greatly decreased numbers of electrons enter and are passed down the ETC, leading to fewer electrons bound by O_2_ to form superoxide.

### AICAR treatment for 48 h increases, but for 10 days decreases, ROS production

Treatment with AICAR for 24 h had little to no effect on ROS production depending upon the exact glucose and pyruvate levels present (Figs 3A,C and 4A,C). However, treatment with AICAR for 48 h almost invariably increased ROS production in both mtDNA mutator and WT MEFs (Figs 3B,D and 4B,D) irrespective of glucose and pyruvate levels. However, after 10 or 20 days of AICAR treatment in HGPUM (Fig. 4E,F), ROS levels were decreased, likely reflecting the lower respiratory rate induced by AICAR at these time points likely as a result of the mechanism described above.

### Rapamycin treatment does not affect the rate of ROS production in WT or mtDNA mutator MEFs

Under almost every culture condition tested, rapamycin treatment had no significant effect on ROS production (Figs 3A-D and 4A-F). The only exception to this was a very small decrease in ROS production in mtDNA mutator MEFs after 24 h of treatment in HGM (Fig. 4A).

### MtDNA mutator MEFs generally show decreased ATP levels in LGM, but increased ATP levels in HGM

**Fig. 5.**
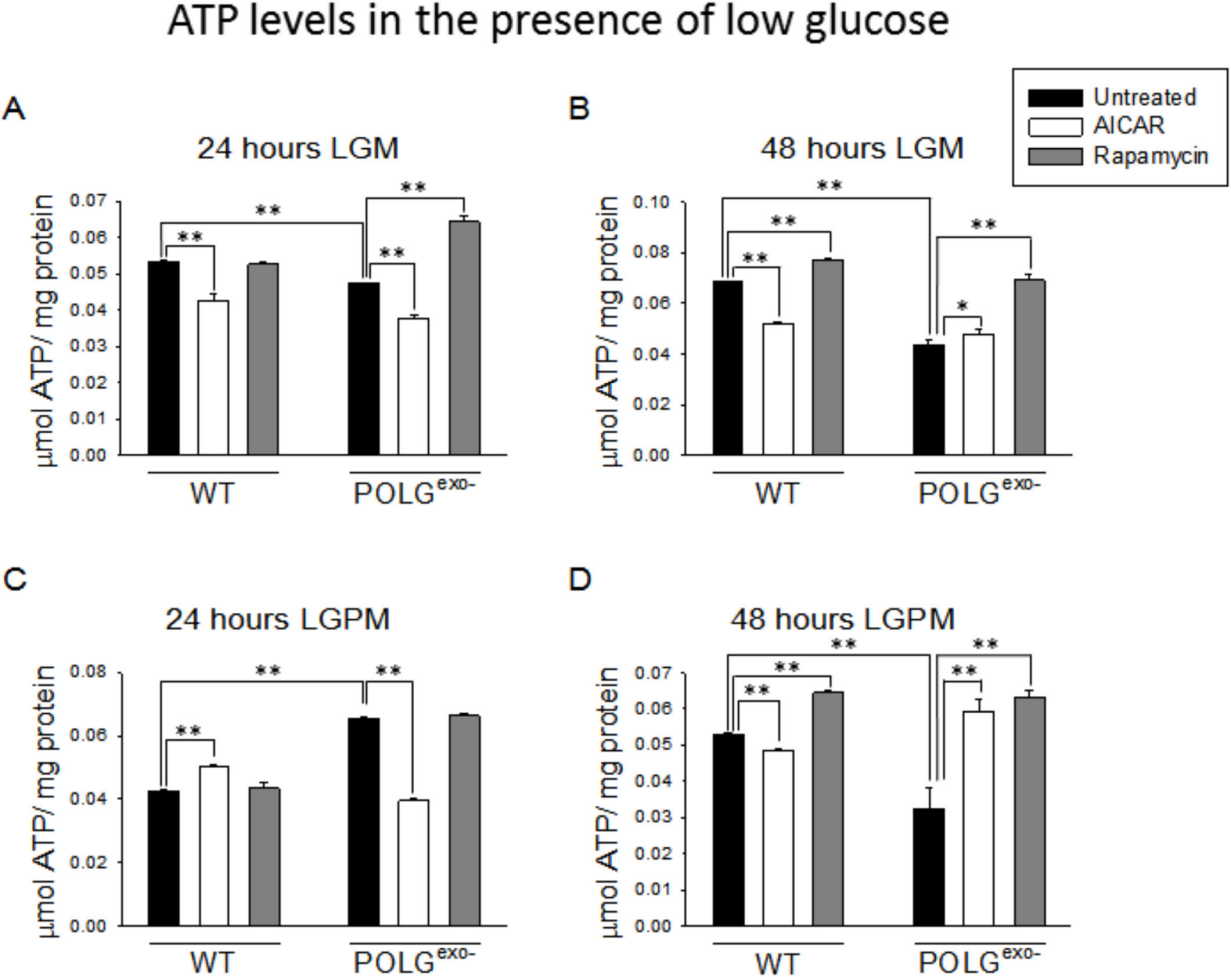
**Effects of AICAR or rapamycin on ATP levels of WT and POLG^exo−^ MEFs under low glucose conditions.** (A) ATP levels after 24 h in LGM. (B) ATP levels after 48 h in LGM. (C) ATP levels after 24 h in LGPM. (D) ATP levels after 48 h in LGPM. Experiments were repeated twice using five technical replicates each time. Bars represent mean±s.e.m. Two-way ANOVA with Fisher's LSD post hoc analysis was performed. *P<0.05, **P<0.001.

**Fig. 6.**
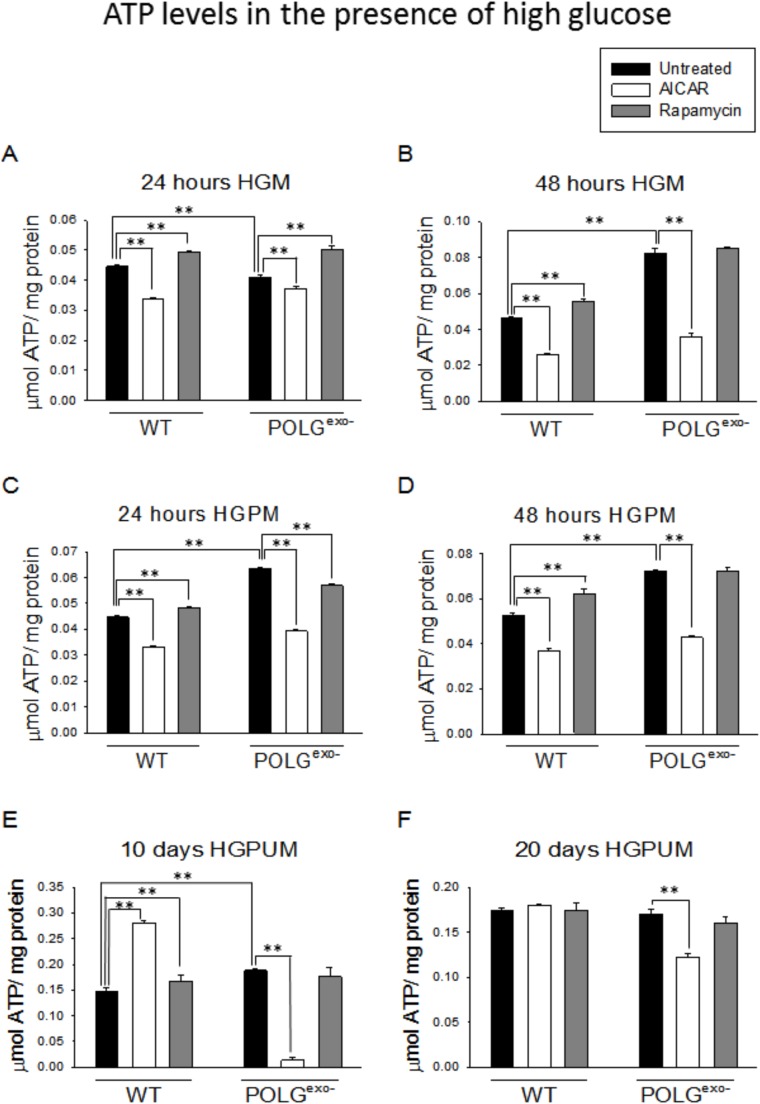
**Effects of AICAR or rapamycin on ATP levels of WT and POLG^exo−^ MEFs under high glucose conditions.** (A) ATP levels after 24 h in HGM. (B) ATP levels after 48 h in HGM. (C) ATP levels after 24 h in HGPM. (D) ATP levels after 48 h in HGPM. (E) ATP levels after 10 days in HGPUM. (F) ATP levels after 20 days in HGPUM. Experiments were repeated twice using five technical replicates each time. Bars represent mean±s.e.m. Two-way ANOVA with Fisher's LSD post hoc analysis was performed. **P<0.001.

We next monitored ATP levels in the mtDNA mutator and WT MEFs. As expected from the results of the respiratory studies, when cultured in LGM or LGPM (Fig. 5A-D), mtDNA mutator MEFs generally showed decreased ATP levels. ATP levels in the mtDNA mutator MEFs dropped significantly between the 24 and 48 h time points as the cells gradually failed to maintain cellular energy levels under the low glucose conditions. But, ATP levels were generally increased in mtDNA mutator MEFs compared with WT MEFs when were they were cultured in HGM, HGPM or HGPUM (Fig. 6A-E). Therefore, there is likely a compensatory upregulation of glycolysis in the mtDNA mutator MEFs that leads to increased ATP levels in the presence of high glucose. To verify this, we measured the rate of acidification of fresh media on confluent cultures grown in HGM. This method is an indirect measure of the rate of glycolysis because the glycolytic end product pyruvate can be reduced to lactate and excreted from cells to acidify the media. As expected, a confluent monolayer of mtDNA mutator MEFs acidified the medium much more rapidly than WT MEFs, with their medium pH being 6.92±0.10 after 48 h and 6.05±0.02 after 72 h, while the WT MEFs' medium pH was 7.24±0.04 after 48 h and 6.84±0.05 after 72 h.

### AICAR treatment decreases ATP levels under most conditions

We next performed experiments treating the WT and mtDNA mutator MEFs with AICAR and measuring cellular ATP levels. When using low glucose culture media, AICAR administration generally decreased ATP levels in WT MEFs and following the 24 h treatment in mtDNA mutator MEFs. But AICAR treatment stabilized ATP levels to completely prevent the drop in ATP levels between the 24 and 48 h time points of the mtDNA mutator MEFs in LGPM (Fig. 5A-D). When cells were cultured in HGM and HGPM, AICAR treatment consistently decreased ATP levels (Fig. 6A-D). This was also true for the 10 and 20 day treatments of mtDNA mutator MEFs in HGPUM (Fig. 6E,F). But in contrast to this, WT MEFs cultured with AICAR for 10 days in HGPUM showed increased ATP levels (Fig. 6E), but this increase dissipated by 20 days of treatment (Fig. 6F).

### Rapamycin increases ATP levels following 48 h of treatment in LGM

When cultured in LGM or LGPM, rapamycin treatment for 24 h (Fig. 5A,C) generally had no significant effect on ATP levels, with one exception. In contrast, when MEFs were cultured for 48 h in LGM or LGPM, rapamycin treatment led to a consistent increase in ATP levels (Fig. 5B,D). When cultured in HGM or HGPM (Fig. 6A-D), the MEFs generally showed a quicker, but greatly blunted response to the rapamycin treatment, especially the mtDNA mutator MEFs, which only showed increased ATP levels at the 24 h time point when cultured in HGM. The WT MEFs showed small increases in ATP levels with rapamycin treatment under all HGM and HGPM culture conditions, as well as at the 10 day time point in HGPUM (Fig. 6E), but this dissipated by 20 days of treatment (Fig. 6F).

### Long term culture of E1A immortalized MEFs in pyruvate-containing media can induce pyruvate addiction

Cell lines with depleted or highly mutated mtDNA can only grow in an HGM with the supplementation of pyruvate and uridine to the culture medium (King and Attardi, 1989). We hypothesized that the immortalized mtDNA mutator MEFs could only accumulate a small amount of mtDNA mutations before cell death when cultured in normal HGM because of the cell's reliance on mitochondrial function under this condition, but a higher level of mitochondrial ETC dysfunction may be revealed if the MEFs were cultured in HGM with pyruvate and uridine (HGPUM) that is permissive for the loss of mtDNA. Results showed that under basal conditions both mtDNA mutator and WT E1A immortalized MEFs formed an average of approximately 30% more colonies when cultured in HGPUM compared to HGM depending upon the specific clonal cell line tested (See day 0 data in Fig. S3). Therefore, the E1A immortalization process appeared to be altering metabolism so that a slight toxicity is induced under the standard HGM culture conditions that was rescued by the presence of pyruvate or uridine in the medium. We next cultured the clonal lines of MEFs for 2-3 months in HGPUM and roughly every 10 days seeded cells into wells on plates containing HGM to determine the number of colonies formed. One E1A immortalized mtDNA mutator MEF line slowly lost the ability to form colonies when placed in HGM over 2 months of culture in HGPUM, while two others did not. Surprisingly, one of three E1A-transfromed WT MEF lines also showed a decreased ability over time to form colonies when taken from HGPUM and seeded into HGM. We also found this slow loss of colony formation to occur when uridine was absent from the media, but not when pyruvate was absent (data not shown), so the decreased rate of colony formation was independent of mtDNA mutations. We decided to call this phenomenon ‘pyruvate addiction’ due to the previously established nomenclature of ‘glucose addiction’ and ‘glutamine addiction’ when describing cancer cells.

In contrast to the results using E1A immortalized MEFs, spontaneously transformed mtDNA mutator cell lines formed colonies equally well in HGPUM and HGM under basal conditions and none of the three spontaneously transformed mtDNA mutator cell lines lost the ability to form colonies in HGM after being cultured for 2 months in HGPUM. Data from one spontaneously transformed clone is shown in Fig. S3. These data suggest that the pyruvate addiction is likely a result of the E1A-based immortalization process.

To learn more about the pyruvate addiction phenotype of the E1A immortalized MEFs, the same types of long term culture experiments were performed as before, but this time the mtDNA mutator MEF line, which previously showed the progressive pyruvate addiction phenotype, was supplemented with compounds known to protect mitochondria or stimulate mitochondrial biogenesis. The cells were then assayed for the ability to form colonies in HGM without the added compound. As shown in Fig. S4, AICAR treatment delayed the pyruvate addiction phenotype, while rosiglitazone, metformin, nicotinamide, resveratrol or a high (50 mM) pyruvate concentration did not. Treatment with a high pyruvate concentration has previously been shown to induce mitochondrial biogenesis (Wilson et al., 2007). AICAR and more specific AMPK activators are known to either increase or decrease the rate of autophagy depending upon the specific cell type studied (Lee et al., 2010; Samari and Seglen, 1998).

In many cases, the rate of autophagy has been reported to positively correlate with organismal lifespan (Hansen et al., 2008; Melendez et al., 2003). Therefore, the effects of two autophagy inducers on pyruvate addiction were tested. Opposite to the results with AICAR treatment, the addition of rapamycin (Fig. S4) or a low 5 mM dose of ammonium chloride (Fig. S5), a concentration that induces autophagy in MEFs (Cheong et al., 2011), greatly stimulated the onset rate of pyruvate addiction. Higher than 10 or 20 mM doses of ammonium chloride – doses that inhibit autophagy in MEFs (Cheong et al., 2011) – were toxic, suggesting that autophagy may be needed for long term survival of the E1A immortalized MEFs. One of the rapamycin-treated pyruvate-addicted clonal cell lines slowly lost the pyruvate addiction phenotype after 2 months in culture and regained the ability to form colonies when cultured in HGM that lacks pyruvate (Fig. S4) demonstrating that pyruvate addiction is a reversible event in a cell population. When E1A immortalized WT MEFs instead of mtDNA mutator MEFs were used and similar results were obtained with rapamycin or 5 mM ammonium chloride addition greatly stimulating pyruvate addiction (Fig. S5).

Since the three autophagy modulators above affected the rate of pyruvate addiction, similar long term experiments were performed supplementing the media with three autophagy inhibitors including 3-methyladenine (a class III PI3K inhibitor), bafilomycin A1 (a vacuolar H^+^ ATPase inhibitor), or chloroquine (a weak base that decreases lysosomal acidity) (Fig. S5). Due to the toxicity of the compounds, concentrations were titrated down from those used in acute studies of autophagy inhibition to allow for long term cell viability (see Materials and Methods for more details). Both 3-methyladenine and bafilomycin A1 affected pyruvate addiction, but the results varied depending upon the specific clonal cell line used, while chloroquine, like AICAR, consistently delayed pyruvate addiction, although to a lesser extent. This data combined with the results from rapamycin and ammonium chloride treatment suggest that autophagy inducers stimulate pyruvate addiction and autophagy inhibitors generally had the opposite effect of delaying pyruvate addiction, although inconsistent results were obtained due to the sub-optimal doses that had to be used to prevent long term toxicity.

Since pyruvate addition increased colony formation in E1A immortalized MEFs in the absence of any added stressor, we hypothesized it would also protect these MEFs against mitochondrial toxins. The addition of pyruvate to the HGM medium (to make HGPM) remarkably increased the IC_50_ of colony formation of mtDNA mutator MEFs in the presence of the complex I inhibitor rotenone from approximately 4 nM to 120 nM (Fig. S6A). The addition of pyruvate also increased the IC_50_ of mtDNA mutator MEFs in the presence of ethidium bromide from roughly 70 ng/ml to 150 ng/ml (Fig. S6B), only a modest increase compared to the IC_50_ of 375 ng/ml measured using WT MEFs in HGM.

## DISCUSSION

In this report it is shown that E1A immortalized mtDNA mutator MEFs can be used as a mitotic cell model to study physiological levels of mitochondrial ETC dysfunction, unlike the non-physiological ETC dysfunction observed in primary MEFs from this premature aging model (Trifunovic et al., 2005). The metabolic effects of potential anti-aging protein kinase modulators and altered levels of energy substrates in the culture media were studied. It was found that AICAR or rapamycin had variable effects on energy metabolism depending upon the culture conditions present, and so therefore, these kinases likely respond to the cellular nutrient availability when regulating energy metabolism. The data produced in this study is complementary to that produced in a study that showed AICAR treatment had greatly different effects on viability, cell growth, mitochondrial biogenesis, ROS production, and mitochondrial membrane potential depending upon the cell line used, with roughly equal numbers of cell lines showing increased or decreased mitochondrial biogenesis and membrane potential following AICAR treatment (Jose et al., 2011).

### Mitochondrial inhibitor sensitivity of WT and mtDNA mutator MEFs

MtDNA mutator MEFs were more susceptible to growth inhibition and toxicity caused by the presence of the mitochondrial toxins FCCP, ethidium, chloramphenicol and azide, but not to the mitochondrial inhibitors antimycin A or oligomycin, or to the oxidizing agents hydrogen peroxide or tert-butyl hydroperoxide. There was also a large trend for increased sensitivity with rotenone. It is not entirely clear why the mtDNA mutator MEFs appeared to be more sensitive than WT MEFs to complex I and complex IV inhibitors, but not to complex III or complex V inhibitors, but it is likely due to the fewer number of genes present in mtDNA that compose complexes III and V. Mammalian mtDNA only encodes one gene for complex III and two genes for complex V, while mtDNA encodes three genes for complex IV and seven genes for complex I. The greater number and size of the genes present in mtDNA increases the likelihood that the genes will be disrupted by mutation in mtDNA mutator cells to sensitize the cells to the toxic effects of the specific ETC complex inhibitors. This rationale also likely explains why the mtDNA mutator MEFs were more sensitive to the mitochondrial protein synthesis inhibitor chloramphenicol, as mitochondrial ribosomal function requires the two large ribosomal RNAs as well as 22 small tRNAs encoded in the mtDNA. Greater apparent susceptibility of mtDNA mutator MEFs to ETC complex I and complex IV inhibitors suggests that complex I and complex IV deficits in the immortalized MEFs are causes for the decreased respiratory rate compared to WT MEFs that occurs when the mtDNA mutator MEFs are cultured in the pyruvate-containing medium. Complex I and complex IV deficits are the primary ETC deficits that occur during aging (Navarro and Boveris, 2007) further highlighting the utility of these cells as an aging model.

The data showing no difference in the toxicity of nucleoside analogs between WT and mtDNA mutator MEFs is consistent with the very slow removal of chain-terminating AZT monophosphate and ddC monophosphate from the end of a DNA strand by the human POLG exonuclease site (Johnson et al., 2001). Although FIAU monophosphate was shown to be a good substrate for POLG exonuclease activity (Johnson et al., 2001), murine mtDNA mutator MEFs did not show increased sensitivity to FIAU toxicity. It is known that FIAU causes liver mitochondrial toxicity in humans, but not in mice (Xu et al., 2014) and this is likely due to a nucleoside transporter targeted to human mitochondria that lacks the mitochondrial targeting signal in mice (Lee et al., 2006). Therefore FIAU may not be efficiently imported into murine mitochondria to affect mitochondrial DNA synthesis.

### E1A immortalized mtDNA mutator MEFs are a novel model to identify therapies for aging-induced mitochondrial dysfunction

E1A immortalized mtDNA mutator MEFs did not show significant mitochondrial respiratory dysfunction in standard HGM culture conditions, but did show respiratory dysfunction compared to WT MEFs when pyruvate was present in the culture medium. However, the E1A immortalized mtDNA mutator MEFs did show increased ROS production under these standard conditions. Both of these phenotypes differ from the results obtained in previous studies of spontaneously transformed mtDNA mutator MEFs where very large declines in respiratory activities occurred under these standard conditions with no increased ROS production (Kukat et al., 2011; Trifunovic et al., 2005). These differences appear to be due to the viral E1A protein used to induce cell immortalization. The exaggerated loss of mitochondrial respiratory function in the primary and spontaneously transformed mtDNA mutator MEFs (Trifunovic et al., 2005) does not replicate the small loss of mitochondrial function commonly observed in aged cells (Beckervordersandforth et al., 2017; Navarro and Boveris, 2007). In addition, the increased ROS production in the E1A immortalized mtDNA mutator MEFs under standard HGM culture conditions make them a desirable cell line for screening for antioxidants, as ROS production increases in most human tissues with age (Navarro and Boveris, 2007) and in almost all aging-related disorders (Gruber et al., 2013). In contrast to the short term studies using HGM, after 10 days of culture in HGPUM mtDNA mutator MEFs showed a decreased respiratory rate and ROS production. Since ROS production can be a signal for mitochondrial biogenesis, these results may explain the decreased levels of mtDNA present in some tissues of mtDNA mutator mice (Safdar et al., 2011).

In rat fibroblasts, expression of the E1A protein was shown to increase mitochondrial gene expression without altering mtDNA levels (Glaichenhaus et al., 1986). This likely occurs through E1A-mediated stabilization of the c-myc transcriptional regulator leading to increased expression of mitochondrial transcription factor A (TFAM) (Chakraborty and Tansey, 2009; Li et al., 2005). The E1A protein is a well-established modulator of the Rb and p53 signaling pathways involved in cellular senescence and apoptosis (Savelyeva and Dobbelstein, 2011), which are activated by Ras signaling (de Groof et al., 2009). In this way, E1A cooperates with activating Ras mutations, such as H-RasV12, in stimulating cell transformation (Seger et al., 2002). In human diploid fibroblasts Ras signaling increased mitochondrial biogenesis and ROS production, which was blocked by the E1A protein (Moiseeva et al., 2009). However, in murine cells such as MEFs, E1A expression did not block Ras-induced mitochondrial biogenesis and Drp1-mediated mitochondrial fission (Serasinghe et al., 2015). In H-RasV12 and E1A transformed MEFs the increased mitochondrial function that occurs following immortalization was lost after around 20 cell passages as Warburg (glycolytic) metabolism was selected (de Groof et al., 2009). The strong Warburg effect in E1A immortalized MEFs likely explains why there is little difference in respiratory rates between the WT and mtDNA mutator genotypes in our study. This high rate of glycolytic function in this model makes these E1A immortalized MEFs a suitable metabolic model of aging in human somatic stem cells that are also mostly glycolytic in energy metabolism (Snoeck, 2015). In contrast, primary MEFs show a more oxidative metabolism.

Moderate mitochondrial dysfunction was found in postmitotic tissues from the mtDNA mutator mice, but mitochondrial dysfunction in mitotic cells and tissues, such as in the blood progenitor cells and intestinal epithelia, was shown to be more severe, likely leading to the mortality of the mice (Kujoth et al., 2005). Others have identified the ability to culture human mitochondrial ETC-deficient fibroblasts in galactose or low glucose media to accentuate mitochondrial dysfunction (Golubitzky et al., 2011; Kukat et al., 2011). These conditions are known to shift cultured cells from a primarily glycolytic metabolic state to a state with higher levels of oxidative phosphorylation, stimulating mitochondrial biogenesis to maintain adequate levels of cellular ATP (Elkalaf et al., 2013). When screening 10 candidate compounds for possible improvement of mitochondrial function in patient ETC complex I-deficient fibroblasts, one group identified AICAR as the most promising compound (Golubitzky et al., 2011). Although the respiratory rate and mitochondrial membrane potential was not increased by AICAR treatment in that study, AICAR treatment increased mitochondrial biogenesis and ATP levels, while improving cell growth and decreasing ROS production.

### Effects of AICAR on the respiratory rate

In the results presented here, an increase in the respiratory rate was not observed in response to AICAR treatment of the immortalized MEFs. Our results are consistent with many other groups that failed to find an increase in the respiratory rate following AICAR treatment (Golubitzky et al., 2011; Guigas et al., 2007; Spangenburg et al., 2013), even though AICAR treatment was shown to induce mitochondrial biogenesis in rodent muscle (Suwa et al., 2015) and human fibroblasts (Golubitzky et al., 2011). Instead, after 10 and 20 days of AICAR treatment in HGPUM, WT MEFs showed a decreased respiratory rate. This decreased respiratory rate may have been caused in part by AICAR-mediated upregulation of pyruvate dehydrogenase kinase 4 (PDK-4) gene expression (Houten et al., 2009), which leads to inhibition of the pyruvate dehydrogenase complex, slowing the conversion of pyruvate to acetyl-CoA in the mitochondrial matrix. AICAR treatment has also been shown to induce many other non-specific effects on cellular metabolism including inhibition of mitochondrial ETC complex I (Guigas et al., 2007) and downregulation of the expression of several mitochondrial ETC genes in a cell line dependent manner (Jose et al., 2011). AICAR as well as the more specific AMPK inhibitor A-769662 have been shown to inhibit oxygen consumption in cell culture, with A-769662 specifically inhibiting respiration in SV40 transformed MEFS (Vincent et al., 2015). AICAR was also shown to inhibit glucose consumption and lactate production by ∼50% in both WT and AMPK-deficient MEFs (Vincent et al., 2015). These effects likely prevented AICAR treatment from increasing respiration, even leading to long term declines in the respiratory rate. An AICAR-mediated decrease in the rate of glucose utilization was also likely responsible for the preservation of ATP levels in the highly glycolytic mtDNA mutator MEFs in LGPM after 48 h, when untreated cells showed decreased ATP, likely from exhaustion of glucose and pyruvate from the media.

Contrary to the sustained positive effects on muscle mitochondrial biogenesis observed during AICAR treatment (Suwa et al., 2015) and more similar to the temporal effects observed here with the E1A immortalized MEFs, increased levels of ETC transcripts were observed in mouse brain during the first week of intraperitoneal injection of AICAR, while expression of these ETC genes was decreased compared to vehicle-injected controls by the day 14 of treatment (Guerrieri and van Praag, 2015). Future studies should determine if long term AICAR treatment decreases markers of mitochondrial mass such as mtDNA levels or citrate synthase activity in specific tissues or cell types.

### Effects of rapamycin on mitochondrial function

The molecular mechanisms behind the increase in respiratory rate of WT MEFs, but not mtDNA mutator MEFs, following the 24 h rapamycin treatment is unknown. However, one possibility is that rapamycin induces a transient, selective increase in mitochondrial uncoupling. mTOR inhibition has been shown to induce partial uncoupling of mitochondrial oxidative phosphorylation in yeast (Pan and Shadel, 2009), rat kidney (Simon et al., 2003; Sivertsson and Friederich-Persson, 2013) and mouse adipose tissue (Polak et al., 2008). Another possible mechanism for increased respiration is increased mitochondrial biogenesis or decreased mitophagy. Mitochondrial biogenesis was observed in adipose tissue following mTOR inhibition (Deepa et al., 2013). A third possibility includes rapamycin transiently inhibiting both mTORC1 and mTORC2 as knockdown of the mTORC2 protein Rictor was associated with increased oxidative metabolism (Schieke et al., 2006). The transient increase in the respiratory rate in WT MEFs 24 h following rapamycin addition in our study was somewhat surprising given that other groups studying myotubes (Cunningham et al., 2007; Ye et al., 2012), leukemic Jurkat T cells (Ramanathan and Schreiber, 2009; Schieke et al., 2006) or T-cell lymphoma (Kittipongdaja et al., 2015) did not find mTOR inhibition to stimulate the respiratory rate and in most cases found a decreased rate of respiration.

mTORC1 activity has been reported to have a significant positive effect on mitochondrial function, increasing the resting respiratory rate and oxidative capacity (Schieke et al., 2006). Rapamycin treatment to inhibit mTORC1 activity has been shown to lower mitochondrial membrane potential, respiratory rate, and ATP synthesis in cultured cells (Schieke et al., 2006). Therefore it was also somewhat surprising that rapamycin increased ATP levels under several culture conditions in this study. However, the increase in ATP levels upon rapamycin treatment is likely due to decreased ATP consumption because of a decreased rate of protein synthesis and other biosynthetic processes as the respiratory rate was generally not changed when ATP levels increased. In addition, inhibition of mTOR has generally been associated with decreased, not increased, glycolytic activity (Sun et al., 2011). Further studies showed that short term (12 h) inhibition of mTORC1 did not affect mitochondrial function (Morita et al., 2013), but longer term inhibition lowered the levels of the transcriptional regulator Yin Yang 1 (YY1), resulting in downregulated expression of PGC-1α and other regulators of mitochondrial function (Cunningham et al., 2007). Knockdown of the mTORC1 protein Raptor decreased the respiratory rate (Schieke et al., 2006). However, it was found that the effects of rapamycin on the respiratory rate of live mice depend upon the duration of rapamycin treatment. Mice treated for 2 weeks showed a decreased respiratory rate, while those treated for 20 weeks showed an increased rate of respiration (Fang et al., 2013). Additionally, rapamycin treatment transiently stimulated mitochondrial biogenesis in the heart of old mice, with markers of mitochondrial biogenesis being upregulated following 2 weeks of administration that returned to basal levels by 10 weeks of administration (Chiao et al., 2016). Therefore, further studies of the temporal effects of rapamycin on mitochondrial function are warranted.

### Pyruvate addiction in E1A immortalized MEFs

The mechanisms through which the E1A protein and autophagy inducers facilitate pyruvate addiction and the mechanisms through which autophagy inhibitors and AICAR decrease the rate of pyruvate addiction are not yet known. Since the respiratory rate slightly increased during the time period of pyruvate addiction, it is hypothesized that treatments that facilitate pyruvate addiction lead to a slow shift in energy production away from glycolysis toward oxidative phosphorylation, resulting in more endogenously produced pyruvate entering mitochondria for energy production and less being used in the cytoplasm by lactate dehydrogenase to maintain cytoplasmic NAD^+^ levels. So, during the time course of pyruvate addiction, the E1A immortalized MEFs likely become reliant on supplemental rather than endogenous pyruvate to provide the cytoplasmic NAD^+^ levels required for normal cell function and division. Since autophagy/mitophagy inducers stimulate pyruvate addiction, it currently appears unlikely that pyruvate addiction is associated with increased mitochondrial mass, but future studies addressing possible relationships between pyruvate addiction and mitochondrial biogenesis or mitophagy may yield important insights into the molecular mechanisms of these processes.

AICAR treatment has been shown to positively or negatively affect the rate of autophagy depending upon the specific cell line or primary cell type tested (Meley et al., 2006; Sanchez et al., 2012), with negative effects mostly reported in hepatocytes and hepatic stellate cells, where AICAR treatment increases mTOR signaling and inhibits TGF-β signaling to inhibit autophagy (Chen et al., 2017). When administered to MEFs, AICAR has been shown to increase the abundance of several upstream markers of autophagy (Lee et al., 2010; Qiang et al., 2013). However, results from a more downstream functional assay of autophagy showed no change in the rate of autophagy in the presence or absence of AICAR (Cheong et al., 2011). AICAR administration has even been shown to increase the pH of lysosomes (Meley et al., 2006), which would decrease the rate of autophagy. Through this mechanism AICAR may be inhibiting autophagy in E1A transformed MEFs to decrease the rate of pyruvate addiction, but further studies are needed to confirm this hypothesis.

To the best of our knowledge this is the first report of a cell line becoming pyruvate addicted, while the study of glucose-addicted or glutamine-addicted cell lines have yielded important insights into human cancer cell metabolism (Wise and Thompson, 2010). Unlike primary murine cells, primary human cells are not immortalized by E1A expression alone and the E1A protein by itself does not stimulate cell division (Li et al., 2004). This may be one reason that pyruvate addiction has not been reported.

### Future perspectives

Recent experiments with mtDNA mutator mice have yielded potential therapies to slow mitochondrial dysfunction with aging. Endurance exercise was shown to rescue the premature aging (Safdar et al., 2011). However, CR, another common anti-aging therapy, was unable to rescue the progeroid phenotype (Someya et al., 2017). The inability of CR to extend lifespan in mtDNA mutator mice suggests that increased mitochondrial ETC function or decreased glycolytic metabolism, either of which can increase NAD^+^ levels, may be required for CR to delay aging. Since rapamycin is a CR mimetic (Nikolai et al., 2015) and CR did not extend the lifespan of mtDNA mutator mice (Someya et al., 2017), rapamycin treatment may not improve mitochondrial function in stem cells, which likely limits the lifespan of these mice. However, rapamycin was able to delay brain pathology in a mouse model of mitochondrial complex I deficiency (Johnson et al., 2013). Future studies should seek to determine the effects of rapamycin on the lifespan of mtDNA mutator mice. In contrast to rapamycin, AICAR has been described as an exercise mimetic (Narkar et al., 2008). Therefore, we predict AICAR to have a better chance at restoring mitochondrial function in stem cells to delay aging in these mice. The exercise-induced rescue of mitochondrial function in mtDNA mutator mice has been suggested to rely upon the mitochondrial function of the p53 protein (Safdar et al., 2016b). Therefore, future studies should focus on the identification of other therapies that can mimic the effects of endurance exercise to stimulate the translocation of p53 to the mitochondrial matrix to increase mtDNA repair.

## CONCLUSIONS

Evidence indicates that mitochondrial dysfunction in stem cells could be a major contributor to aging and age-related diseases, underscoring the need for mitotic cell models of mitochondrial dysfunction to conveniently study this phenomenon. E1A immortalized mtDNA mutator MEFs were created to fill this void. Under the standard HGM conditions used, they did not show respiratory dysfunction, but did show increased ROS production, both of which are contrary to results obtained using the parental primary MEFs. However, upon pyruvate addition to the media, immortalized WT MEFs showed greater respiration than the immortalized mtDNA mutator MEFs. Rapamycin treatment of WT MEFS resulted in a transient increase in respiration that did not occur following treatment of mtDNA mutator MEFs. In both mtDNA mutator and WT MEFs under high glucose conditions short term AICAR treatment increased ROS levels and decreased ATP levels, which in WT MEFs upon a longer duration of treatment transitioned into decreased respiratory rates and ROS levels. Short term AICAR treatment increased ATP levels when ATP levels were low and generally decreased ATP levels when ATP levels were high. The future use of E1A immortalized mtDNA mutator MEFs and other cell lines containing active p53 should facilitate research into the role of mtDNA mutations in stem cell aging and the mechanisms responsible for the protective effects of endurance exercise on animals with high levels of mtDNA mutations.

## MATERIALS AND METHODS

### Generation of stable cell lines

MEFs were obtained from WT and homozygous POLG^D257A/D257A^ (POLG^exo−^) mitochondrial DNA mutator mouse decapitated embryos (Kujoth et al., 2005) according to standard protocols and frozen down (Lei, 2013). To generate stable cell lines, the MEFs were thawed, plated and then stably transfected with a plasmid containing the adenoviral E1A gene and a puromycin selectable marker using a retroviral transfection system (Swift et al., 2001). E1A-induced immortalization was used instead of spontaneous transformation to preserve contact inhibition and monolayer growth and in an attempt to preserve the function of genes in signaling pathways that commonly mutate during spontaneous transformation. E1A immortalized MEFs also formed well-defined colonies that were easier to count in toxicity assays than spontaneously transformed MEFs. We, like others (Kukat et al., 2011), found a higher rate of spontaneous transformation of the mtDNA mutator MEFs compared to the WT MEFs (data not shown). In rare instances following several months of culture some lines of E1A immortalized MEFs would lose the ability to proliferate, flatten and appear to undergo cellular senescence. In general cells appeared healthy, but no stringent check was performed for cell line contamination.

### Cell culture

WT and mtDNA mutator MEFs were grown in either 96-well plates or in 10 cm culture dishes using HyClone^®^ Dulbecco's modiﬁed Eagle's medium (DMEM) supplemented with 10% FBS, 30 µg/ml penicillin, 50 µg/ml streptomycin, and 2 mM L-glutamine (Gibco; Thermo Fisher Scientific). For short term studies, cells were grown using either high (4.5 g/l) or low (1 g/l) glucose DMEM with or without 1 mM pyruvate for 24, 30 or 48 h. For longitudinal studies, high glucose media with 1 mM pyruvate and 50 µg/ml uridine (HGPUM) was used. Longitudinal (10, 20 and 29 days of treatment) studies were only performed in HGPUM since MEFs cultured in low glucose media for the measurement of oxygen consumption frequently did not survive the trypsinization step prior to placing the cells in the Clark electrode chamber. The media was changed every 2 days for cells grown for the 10, 20 and 29 day longitudinal studies. For the respiratory, ROS production and ATP measurements, cells were treated with 1 mM AICAR or with 10 nM rapamycin (LC Labs).

### ATP assays

CellTiter-Glo (Promega, Madison, USA) ATP detection reagent was used to determine the levels of ATP following a 10 min 22°C incubation of cells with CellTiter-Glo reagent. CellTiter-Glo reagent was combined with the cell suspension in a 1:1 ratio in a 96-well microplate and then shaken for 2 min. Luminescence was read using a Biotek Synergy 2 microplate reader after the contents of the 96-well plate were transferred to a white opaque bottom plate for increased signal.

### Oxygen consumption analysis

Cells were trypsinized from 10 cm dishes, the trypsin was deactivated with culture media, and the cells were spun down and resuspended in 1 ml of the appropriate fresh culture media. 350 µl of the suspension was then placed into a Strathkelvin Mitocell MT200A respiratory chamber with a Clark type electrode at 37°C with a stir bar. The cellular respiratory rate was recorded for 4 min. The middle 1 min was chosen for slope analysis. Oxygen consumption was normalized to the total cell number in the chamber. Cell counts were obtained using a hemocytometer. Both the mtDNA mutator and WT MEFs greatly lost the ability to respire upon trypsinization when cultured in low glucose culture medium for 36 h or longer, and so time points for oxygen consumption analysis were not obtained beyond 30 h when the MEFs were cultured in this medium.

### ROS measurements

2′,7′-dichlorodihydrofluorescein diacetate was used to detect ROS production. Following hydrolysis of the acetate esters by intracellular esterases, dichlorodihydrofluorescein is produced. Dichlorodihydrofluorescein, when oxidized by ROS, becomes the highly fluorescent compound dichlorofluorescein (DCF). A 2′,7′-dichlorodihydrofluorescein diacetate stock solution of 500 µM was made up in 2% DMSO in PBS and the tube was wrapped in foil to prevent exposure to light. Media was aspirated from the cells grown in 96-well plates and the cells were washed once with 100 µl PBS. An assay concentration of 50 µM was obtained by mixing 10 µl of the stock solution with 90 µl of PBS and added to the monolayer of cells. As a positive control, 0.5 µl of denatured 100% EtOH was added, as it can greatly stimulate mitochondrial ROS production at electron transport chain complexes I and III (Cunningham and Bailey, 2001). The plate was incubated in the dark at 37°C for 30 min. The fluorescence was then measured using a Biotek Synergy 2 microplate reader with an excitation filter of 485/20 nm and emission filter of 528/15 nm. Values were normalized to the total amount of protein in each well.

### Protein assays

Cells were lysed and extracts were solubilized using modified RIPA buffer without SDS. A determination of the protein concentration was performed using the BCA protein assay (Pierce; Thermo Fisher Scientific). Results were normalized using the protein concentrations present for the assays.

### Colony formation drug toxicity assays

The E1A immortalized MEFs were cultured in HGM consisting of high glucose DMEM with the addition of 10% fetal bovine serum, penicillin (100 U/ml), streptomycin (100 mg/ml) and L-glutamine (2 mM). Three distinct mtDNA mutator MEF clones and three distinct WT MEF clones were used for each experiment. 2000 cells were placed in each well of a 6-well plate in HGM. For each 6-well plate, five different concentrations of a drug were added 1 day after the cells were plated, leaving one untreated well per plate. Drug concentrations were doubled or tripled from one well to the next depending upon the specific compound tested. Cells were grown for 8-10 days with one change of media. Then the culture media was removed and the cells were washed twice with PBS. A 5% crystal violet stock solution was made in 95% ethanol. 500 ml of a 0.5% crystal violet staining solution was made by combining 50 ml of the 5% crystal violet stock solution with 350 ml of methanol, 50 ml of 35% formaldehyde and 50 ml of deionized water. The cells were fixed and stained for 10 min. Then the solution was removed and the wells were washed several times with deionized water until the colonies became easily visible, the wells were allowed to dry, and the colonies were manually counted with a colony counting pen. The IC_50_ is the compound concentration that allows half the maximal number of colonies to grow. The IC_50_ values were calculated from the dose response data using GraphPad Prism software.

### Assays for determining compounds that alter the rate of pyruvate addiction

The E1A immortalized MEFs were cultured in triplicate in 6-well plates using HGPUM consisting of high glucose DMEM media supplemented with 10% fetal bovine serum, penicillin (100 U/ml), streptomycin (100 mg/ml), L-glutamine (2 mM), pyruvate (1 mM), uridine (50 µg/ml) and a compound to be tested. Several autophagy inhibitors including 3-methyladenine, bafilomycin A1, chloroquine and ammonium chloride were toxic to the cells at the normal doses used to inhibit autophagy, so concentrations were titrated down to allow for long term viability. For example, a typical acute treatment dose of 3-methyladenine is 5 mM, but for long term treatment the immortalized MEFs could only survive at a concentration of 4 mM. Typical acute treatment doses of bafilomycin A1 for autophagy inhibition are 100 nM-1 µM, but the immortalized MEFs could only survive long term at a concentration of 1 nM. Typical acute treatment doses of chloroquine range from 10-100 µM, but the immortalized MEFs could only survive long term at a concentration of 6 µM. Typical acute concentration doses of ammonium chloride used for autophagy inhibition are 10-20 mM, but the immortalized MEFs could only survive long term at a concentration of 5 mM. We found AICAR to be toxic at a concentration of 3 mM, so we used a 1 or 2 mM dose. We repeatedly found that following rapamycin treatment for 8-12 days, the vast majority of the cell population died suddenly, possibly due to delayed mTORC2 inhibition (Barlow et al., 2012), but we continued the long term culture with the cells that were selected to resist this toxicity.

Every 3-4 days, cells would become confluent and so were washed in PBS and trypsinized. Ninety per cent of the cells were discarded and the remaining 10% of the cells returned to the same well for continued culture. Every 9-12 days the 90% of the cells that were normally discarded were counted using a Coulter Z1 Particle Counter and 2000 cells from each well were seeded into a well in each of two new 6-well plates. One 6-well plate was cultured using HGPUM without drug treatment and the second 6-well plate was cultured in the same media lacking pyruvate and uridine (HGM). Cells were grown for 8-10 days with one change of media. Culture media was removed and the cells were washed twice with PBS, and fixed and stained with 0.5% crystal violet staining solution as described above, de-stained with deionized water, and colonies were counted manually. The percentage of the number of colonies that formed in the absence of pyruvate and uridine (HGM) compared to the number of colonies that formed in the presence of pyruvate and uridine (HGPUM) was plotted each 9-12 days for 3 weeks to 3 months depending upon the rate of the loss of colony formation of the cells seeded in media lacking pyruvate and uridine (HGM).

### Statistical analysis

SigmaPlot version 11.0 was used for statistical analysis and graph generation. For most of the analyses two-way ANOVA tests were performed with genotype and treatment variables followed by Fisher's LSD post hoc means comparison. Unpaired *t*-tests were used for the colony counting assays.

## Supplementary Material

Supplementary information
