# Supplementary material for: The effects of AICAR and rapamycin on mitochondrial function in immortalized mitochondrial DNA mutator murine embryonic fibroblasts
Source: Biol Open. 2018 Sep 3;7(11):bio033852. doi: 10.1242/bio.033852 (PMC6262855; doi:10.1242/bio.033852)
Supplement: Supplementary information [file biolopen-7-033852-s1.pdf]

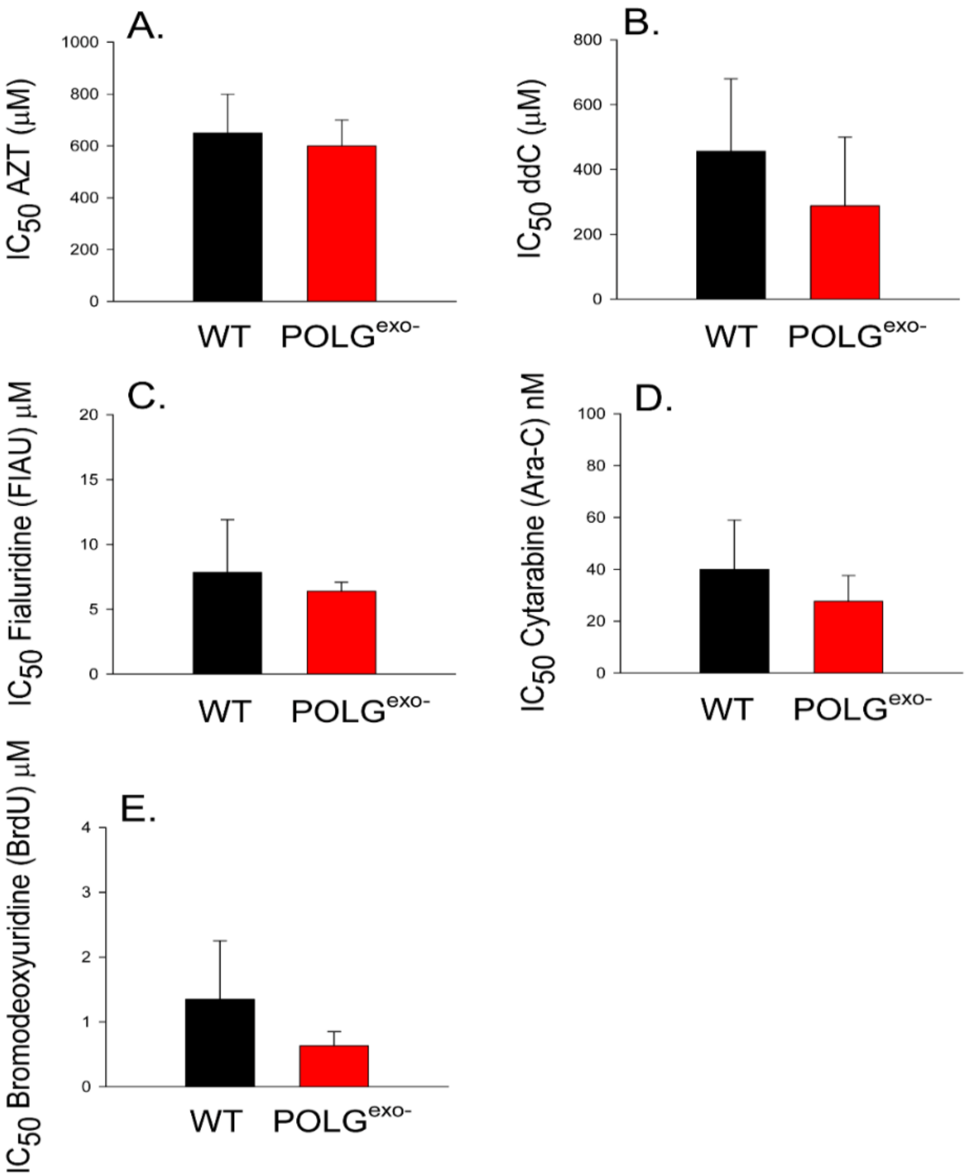

Fig. S1. MtDNA mutator (POLG^{exo-}) MEFs do not show increased sensitivity to the toxicity of nucleoside analogs. A colony counting assay was performed to determine IC_{50} values for the nucleoside analogs. The nucleoside analogs tested were (A) AZT, (B) ddC, (C) FIAU, (D) Ara-C, and (E) BrdU. Experiments were performed with 3 independently derived E1A immortalized WT and mtDNA mutator clone lines. Unpaired t-tests were used for data analysis.

Biology Open • Supplementary information

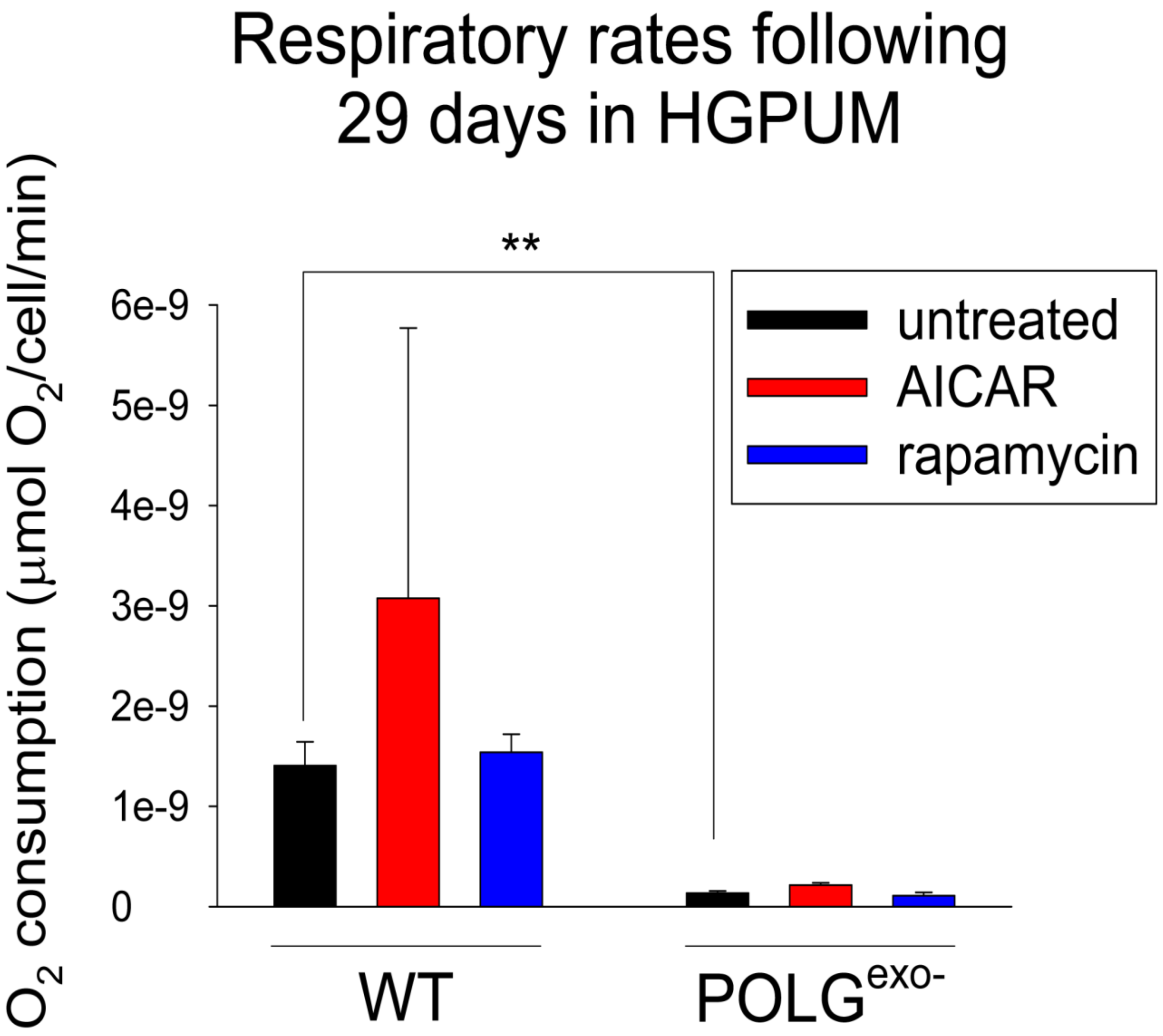

Fig. S2. AICAR or rapamycin treatment for 29 days in HGPUM did not significantly alter the rate of O_2 consumption. Untreated mtDNA mutator ($POLG^{exo-}$) MEFs showed a lower rate of oxygen consumption than untreated WT MEFs (** $p < 0.001$). Experiments were repeated twice using 3 technical replicates each time (HGPUM = high glucose pyruvate and uridine medium). Two-way ANOVA with Fisher's LSD post hoc analysis was performed for data analysis.

Biology Open • Supplementary information

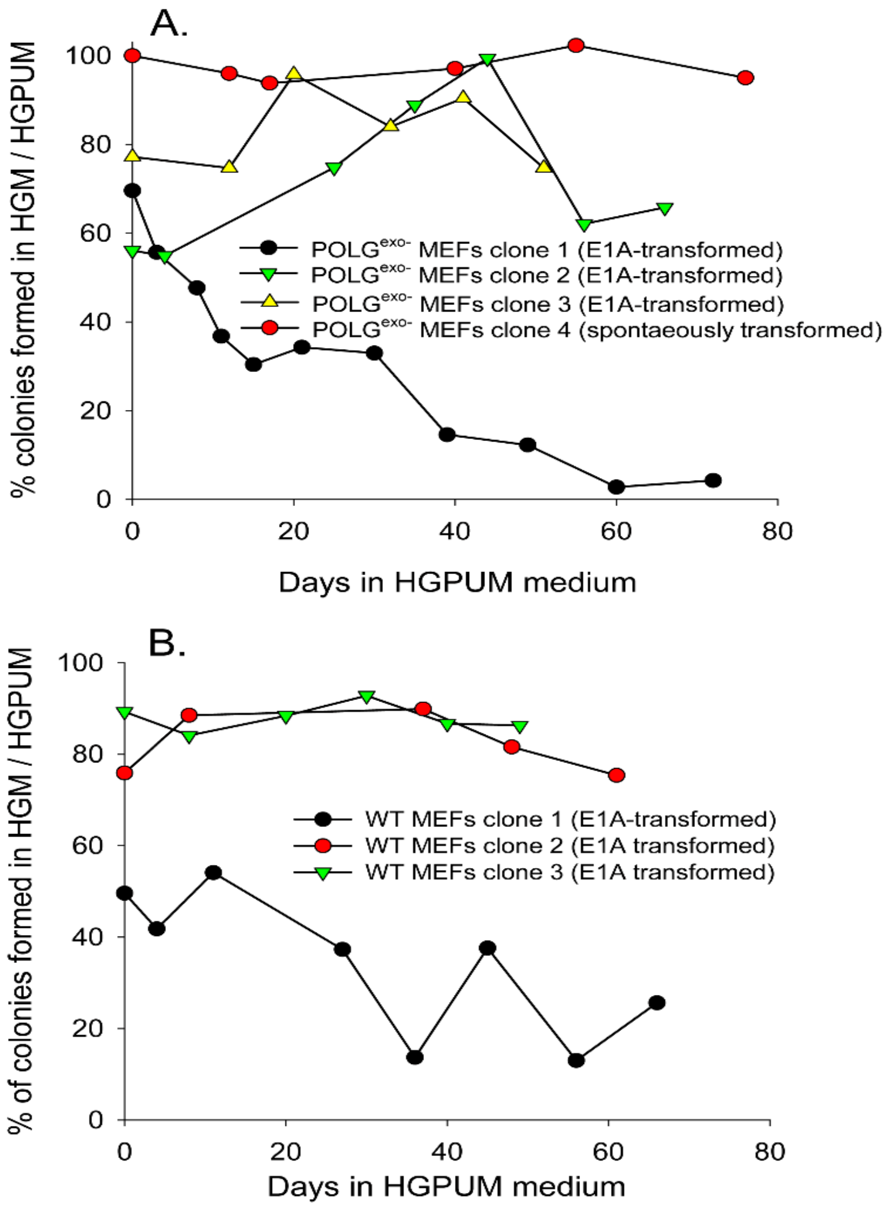

Fig. S3. Select E1A immortalized MEF clones become addicted to pyruvate when cultured long term in HGPUM. (A) MtDNA mutator (POLG^{exo-}) MEFs (B) WT MEFs. Roughly every 10 days equal numbers of cells were seeded in both HGM and HGPUM media and the percentage of cells that form colonies in HGM compared to those that form colonies in HGPUM was plotted. Experiments were performed with the 3 independently derived E1A immortalized WT and mtDNA mutator clone lines.

Biology Open • Supplementary information

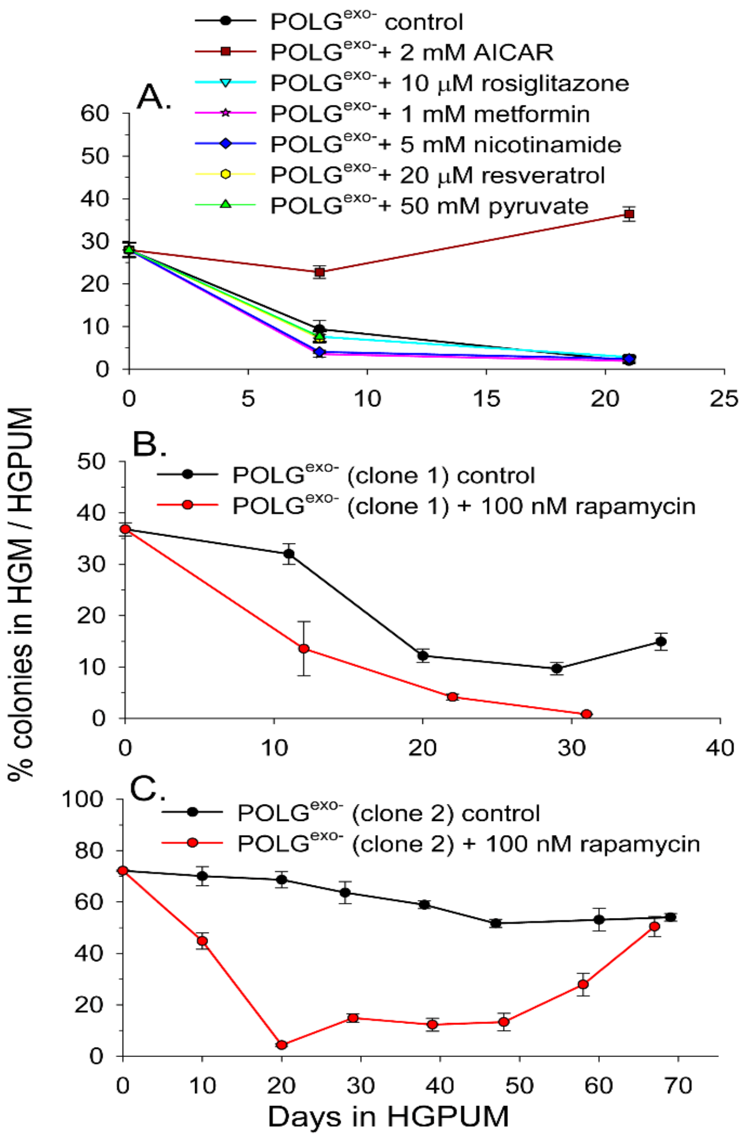

Fig. S4. AICAR treatment delays pyruvate addiction, while rapamycin treatment stimulates it. Colony counting assays were performed following long term growth of the mtDNA mutator (POLG^{exo-}) MEFs in HGPUM in the presence of (A) AICAR, rosiglitazone, metformin, nicotinamide, resveratrol, or pyruvate at the concentration indicated (POLG^{exo-} clone 1). AICAR delayed pyruvate addiction (t-test, $p < 0.001$). (B) Colony counting assays were performed in the presence of 100 nM rapamycin (POLG^{exo-} clone 1) or (C) in the presence of 100 nM rapamycin (POLG^{exo-} clone 2). Rapamycin stimulated pyruvate addiction (t-test, $p < 0.001$). Experiments were performed using 3 technical replicates.

Biology Open • Supplementary information

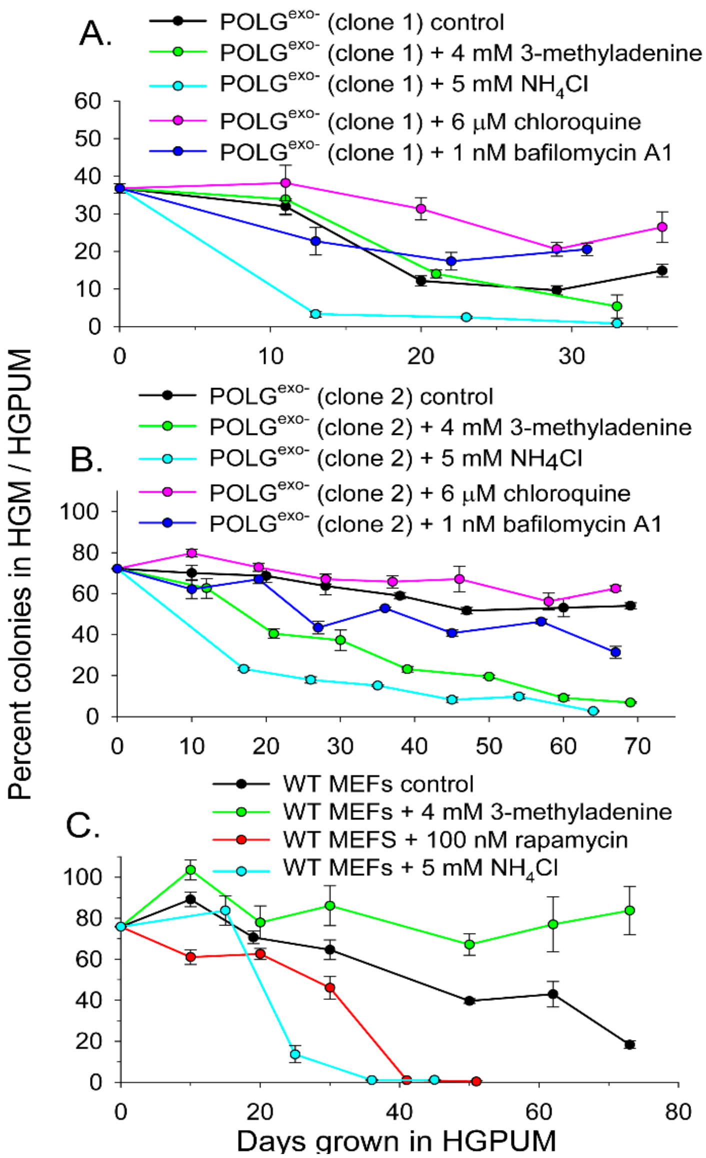

Fig. S5. Ammonium chloride (NH_4Cl) treatment stimulates pyruvate addiction while chloroquine treatment delays it. Colony counting assays were performed following long term growth in HGPUM in the presence of 4 mM 3-methyladenine, 5 mM ammonium chloride (NH_4Cl), 6 µM chloroquine, 1 nM bafilomycin A1, or 100 nM rapamycin. (A) mtDNA mutator ($POLG^{exo-}$) MEFs (clone 1) were used. (B) mtDNA mutator ($POLG^{exo-}$) MEFs (clone 2) were used. (C) WT MEFs were used. Experiments were performed using 3 technical replicates. NH_4Cl stimulated pyruvate addiction in every clone tested (t-test, $p < 0.001$). Chloroquine delayed pyruvate addiction in both mtDNA mutator clones tested (t-test, $p < 0.001$).

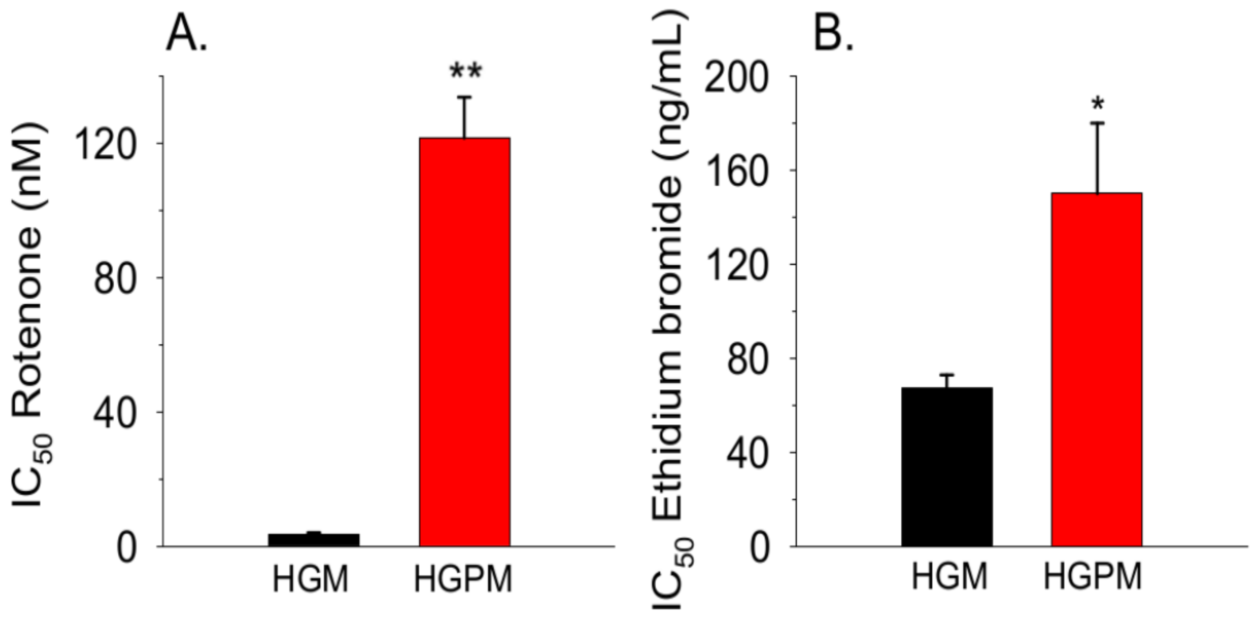

Fig. S6. Pyruvate protects E1A immortalized mtDNA mutator MEFs from the toxicity of rotenone or ethidium bromide. 1 mM sodium pyruvate was present in the HGPM medium. Colony forming assays using E1A immortalized mtDNA mutator MEFs in the absence or presence of 5 different concentrations of rotenone or ethidium bromide were performed to determine IC_{50} values as described in the methods. A) The presence of pyruvate in the HGPM increased the IC_{50} of rotenone (** t-test, $p < 0.001$). B) The presence of pyruvate in HGPM increased the IC_{50} of ethidium bromide (* t-test, $p < 0.05$). Experiments were performed using 3 independently derived E1A immortalized mtDNA mutator clone lines (*n*=3).

Biology Open • Supplementary information
